# Gaps and Gains in Informed Consent for Surgery in a Non-Western Context: Beyond the Signatures of Iranian Patients

**DOI:** 10.1007/s41649-025-00372-2

**Published:** 2025-11-17

**Authors:** Malihesadat Taghriri, Maryam Modabber, Alireza Parsapour, Shirzad Nasiri, Ayat Ahmadi, Ehsan Shamsi Gooshki

**Affiliations:** 1 Medical Ethics and History of Medicine Research Center, Tehran University of Medical Sciences, Tehran, Iran; 2 Department of Surgery, Shariati Hospital, School of Medicine, Tehran University of Medical Sciences, Tehran, Iran; 3 Knowledge Utilization Research Center, Tehran University of Medical Sciences, Tehran, Iran; 4 Monash Bioethics Center, Faculty of Arts, Monash University, Melbourne, Australia

**Keywords:** Informed consent, Medical ethics, General surgery, Iran

## Abstract

Informed consent is essential for protecting patients’ rights and ensuring ethical medical practices. This study aimed to evaluate the quality and identify deficiencies in the informed consent forms of surgical patients at Shariati Hospital in Tehran. This descriptive cross-sectional study, conducted in 2022, reviewed the medical records of 288 surgical patients at Shariati Hospital from 2017 to 2021. A researcher-made checklist assessed demographic data, disease specifics, informed consent information, and completeness. Deficiencies in the forms and patients’ understanding were evaluated using a Likert scale, with checklist validity confirmed by a pilot study (Cronbach’s alpha = 0.75). Data analysis was performed using SPSS v.24. This study of 288 patient records found that while 93.4% had consent forms, only 2.2% were adequately informative and 4.5% understandable. Wound infection was the most discussed complication (89.6%), with no significant link to surgery type. Third-party consent was rare (2.8%), and 83% of forms had witnessed signatures. Residents obtained 98.5% of consents. This study identifies significant gaps in the informed consent process at a hospital, particularly in the disclosure and understanding of risks, benefits, and alternatives. While it does not assess voluntariness or decision-making capacity, it shows positive trends, such as increased healthcare team involvement and reduced third-party authorization, especially for female patients. The findings highlight a shift toward legal protections over ethical considerations, with implicit safeguards, like fingerprints and witness signatures, replacing explicit waivers.

**Supplementary Information:**

The online version contains supplementary material available at 10.1007/s41649-025-00372-2.

## Introduction

Informed consent (IC) stands as a cornerstone of ethical medical practice, weaving the bioethical principle of “autonomy” into the fabric of patient care by empowering individuals to exert control over their bodies and medical choices, a concept rigorously championed in contemporary scholarship (Rao 2008; Katz 1977; Beauchamp 2011). However, other ethical principles could be used to argue for the significance of IC, such as beneficence and non-maleficence. This principle, central to contemporary bioethics, is a crucial aspect of the patient-physician relationship, which insists that patients should not be mere passive recipients of care but active participants in their treatment, realizing their agency as autonomous moral agents. This marks a significant shift from historical paternalism, where physicians solely dictated treatment decisions (Faden and Beauchamp 1986; Beauchamp and Childress 1994). The status of IC in contemporary medicine and bioethics is also related to the fundamental global social, cultural, and political changes, e.g., civil rights movements and the establishment of democracies, especially during the twentieth century. The move toward a patient-centered model reflects the emergence of patient-centered care and “shared decision-making,” a collaborative process rooted in mutual respect and open dialogue. However, the successful implementation of this approach depends heavily on cultural and systemic factors (Beauchamp 2011; Faden and Beauchamp 1986; Jørgensen and Rendtorff 2018).

Despite the fact that informed consent is a concept mostly recognized and utilized in the second half of the twentieth century and gained significant attention after the post-World War II Nuremberg trials in response to the involvement of Nazi doctors in unethical and coercive medical experiments, the history of seeking consent from patients carries a rich historical tapestry that extends beyond its commonly cited origins in post-World War II Western ethical codes, finding resonance in earlier non-Western traditions. One example is the presence of a similar concept in Islamic tradition in texts such as the seventieth-century text “*Bihar al-Anwar*” by *Majlisi*, who underscores the profound significance of securing a patient’s voluntary agreement to treatment, weaving an early thread of moral insight that anticipates the contemporary emphasis on informed acquiescence (Allama Muhammad Baqir Majlisi 1981). This longstanding tradition in the Islamic tradition highlights an ethical commitment to respecting patients’ choices that spans different cultures than Western liberal democracies, demonstrating an instance of the enduring importance of patient agency in influencing medical practices in other cultures.

IC is essential for most medical procedures and serves as a fundamental aspect of clinical practice. Its significance is particularly heightened in surgical settings, where it empowers patients to make independent decisions prior to undergoing invasive procedures that involve anesthesia, costs, and extended recovery times (Basukala et al. 2023; Hammami et al. 2014). In contemporary medicine, IC is not only an ethical obligation and moral commitment of healthcare professionals, but it is also supported by legal systems in many jurisdictions, which overriding it could end in serious legal sanctions.

The implementation of IC highlights a significant gap between ethical principles and legal requirements. Legally, it often functions as a mere administrative formality that is fulfilled with a signature, serving primarily to ensure compliance before elective surgeries. Ethically, however, IC calls for something far richer: a patient’s deep comprehension and freely given consent, rooted in the principle of autonomy, which honors their right to shape their own fate, and beneficence, which seeks their well-being through a transparent unveiling of risks and hopes (Beauchamp 2011; Hall et al. 2012; Paterick et al. 2008; Parsapour et al. 2005). This disconnect—where a signed form may fulfill legal requirements but still fail to respect patient autonomy—presents significant ethical challenges. It risks turning an important moral safeguard into just a bureaucratic procedure, which can erode trust and diminish patients’ sense of agency. Additionally, these differences, influenced by social, religious, and political factors, highlight the urgent need for a thoughtful and culturally sensitive approach to IC (Hall et al. 2012).

The IC process, which is a crucial component of standard care, has been originally shaped in the twentieth century by international research ethics guidelines such as the Nuremberg Code and the Declaration of Helsinki and very soon emphasized and further guided to be implemented in clinical medicine by the World Medical Association’s main medical ethics documents, including the Declaration of Geneva and the International Code of Medical Ethics, as well as policies from the World Health Organization. Standard IC requires careful evaluation of patients’ decision-making capacity and voluntariness, disclosing relevant information such as describing the intervention, reviewing available alternatives, disclosing potential risks and benefits, ensuring patients’ correct understanding of the medical procedure, and finally obtaining patient preferences and documentation of consent in informed consent forms (Hall et al. 2012; Shah et al. 2024; Leclercq et al. 2010; Schenker and Meisel 2011). Consent forms should typically outline both general and specific risks, the implications of not receiving treatment, alternative options, and clarify that outcomes cannot be guaranteed in plain language which could be easily understood by patients. This comprehensive approach allows patients to thoughtfully consider the reasons, methods, risks, benefits, and choices involved in making informed decisions (Spatz et al. 2016). The amount and quality of information expected to be mentioned in informed consent forms vary, and there are different standards for determining what should be disclosed. However, the most famous and widely used standard is the reasonable person standard. Despite its widespread use, this standard has been criticized for being vague and lacking clear guidance on how much information is necessary to meet its requirements (Greenblum and Hubbard 2019).

Obtaining IC is recognized as a fundamental practice in Iran. This principle is reflected in various legal, medical, and religious documents, including the Islamic Penal Code, the Patients’ Rights Charter, the Code of Ethics for Medical Professionals of the Medical Council of Iran, and the Accreditation Guidelines for Iranian Hospitals (Parsapoor et al. 2014; Shamsi-Gooshki et al. 2020; “The Constitution Law of the Islamic Republic of Iran, Article 158, 495.” 1991). Despite this formal acknowledgment, there are significant gaps in practice, as empirical evidence shows that consent forms are often incomplete and requirements for ensuring patients’ understanding are frequently overlooked. Despite the recognition of individual informed consent in the Iranian context, in real-life medical practice, the IC process is heavily influenced by cultural and familial dynamics that make it challenging. In many cases, families act as intermediaries in decision-making, reflecting a collectivist approach that can diminish the patient’s self-determination, particularly for women who often face additional sociocultural challenges in asserting their autonomy (Jarayedi and Asghari 2018; Kim 2009; Shaibu 2007). This stands in stark contrast to accepted practices, where IC typically emphasizes direct patient involvement with minimal third-party influence (Shaibu 2007). Additionally, a unique aspect of the consent process in Iran is the common practice of including witnesses—usually family members—who help verify the consent process, ensuring familial oversight and ethical accountability. This contrasts with the West, where such roles are not typically seen (Pai et al. 2023).

Research highlights significant deficiencies in the IC process in Iran. A 2014 study revealed that the information provided to patients was markedly inadequate (Faghanipour et al. 2014). Similarly, a 2017 study found that the quality of obtaining informed consent remained substantially below standard (Joolaee et al. 2017). Adding to these challenges, IC is often obtained just minutes before surgery, a time when patients are least equipped to process complex information, which further undermines their autonomy (Shahu et al. 2017). Genuine patient independence—the ethical foundation of IC—relies on clear and thorough disclosure. However, pressures for efficiency frequently reduce the process to a mere formality focused on obtaining a signature (Hall et al. 2012; Basukala et al. 2023).

These challenges arise partly from insufficient training for physicians and a systemic neglect of patients’ rights to ask questions or seek clarification (Hudak et al. 2008). The consequences of these shortcomings can be significant, leading to decreased patient satisfaction, non-compliance with treatment, regret, and an increase in complaints against healthcare providers (Schuler et al. 2019; Najari et al. 2020; Tanno and Bito 2019).

To improve the IC process, it is vital to identify these barriers and implement necessary changes promptly. Therefore, this study aims to evaluate the quality of IC at Shariati Hospital in Tehran from 2017 to 2021, identify existing deficiencies, and propose reforms to enhance patient-provider interactions and reduce complaints.

## Methods

This research was designed and conducted as a descriptive cross-sectional study in 2022. The aim was to identify the flaws and deficiencies in IC forms and to provide guidance to medical professionals and stakeholders on how to obtain IC. The research data comprised the medical records of surgical patients in the General Surgery Department, which were retrieved from the Hospital Information System (HIS) of Shariati Hospital in Tehran from 2017 to 2021. A stratified sampling method with proportional allocation was used for sample selection. Considering an undesirable quality ratio of 0.25, a Type I error of 0.05, and a margin of error of 0.05, the required sample size was calculated to be 288 cases. The inclusion criteria for the study involved the medical records of patients who underwent various surgeries, including thyroidectomy, open appendectomy, laparoscopic appendectomy, open herniorrhaphy, laparoscopic herniorrhaphy, open cholecystectomy, and laparoscopic cholecystectomy.

To collect data, a researcher-made checklist was utilized. In constructing this checklist, the researcher first reviewed various scientific resources and studied the most recent and relevant literature and guidelines regarding the elements of IC and information related to ten surgical procedures. Appropriate items were gathered and extracted from different scientific sources and guidelines. Additionally, to complete the items, feedback from professors and specialists in surgery and medical ethics was obtained during two expert panel sessions. Ultimately, seven surgical procedures were selected, and for each procedure, between 56 and 74 items were prepared. The variation in the number of items was due to the different numbers of specific complications, alternative treatments, and potential risks associated with the various surgeries. The checklist for each of the seven surgical procedures was reviewed by two professors, and to assess the validity of the checklist, a pilot study was conducted with ten records from the selected surgeries. The reliability of the checklists was also confirmed using internal consistency and the calculation of Cronbach’s alpha (*α* = 0.75).

The items in the checklist were designed in four sections in accordance with the study objectives. The first section included demographic information about the patients, such as age, gender, marital status, and place of residence. The second section pertained to disease-related information, including the type of disease and duration of hospitalization. The third section included an assessment of the general information in the IC form, containing items such as the existence of a consent form in the patient’s file, the mention of the patient’s full name in the informed consent form, the date and time noted by the consent provider, the relationship of the consent provider to the patient, the method of confirming the consent provider’s identity (signature or fingerprint), the method of confirming the witness’s identity (signature or fingerprint), the title/position of the witness, the name of the individual obtaining consent, the title/position of the individual obtaining consent, the method of confirming the consent provider’s identity, the date and time recorded by the consent provider, the presence of forensic medicine confirmations, obtaining spousal consent, the presence of any strike-throughs in the IC form, the mention of the reason for not obtaining consent from the patient (obtaining consent from someone other than the patient), and the presence of psychiatric consultation to inform or ensure the decision-making capacity of the patient.

The fourth section of the study consisted of three parts. Part “a” focused on providing information about the surgical procedure, and evaluating the understandability of this information is for a layperson. This was assessed using a Likert scale from 1 to 5: 5 for “completely acceptable,” 4 for “acceptable,” 3 for “intermediate,” 2 for “somewhat acceptable,” and 1 for “unacceptable.” Additionally, it assessed the completeness of information regarding the benefits of the surgery using the same five-point Likert scale: 5 for “completely comprehensive,” 4 for “comprehensive,” 3 for “intermediate,” 2 for “unacceptable,” and 1 for “completely unacceptable.” Part “b” addressed the general and specific complications of each specific surgical procedure (thyroidectomy, open appendectomy, laparoscopic appendectomy, open herniorrhaphy, laparoscopic herniorrhaphy, open cholecystectomy, and laparoscopic cholecystectomy) and evaluated the completeness of the information regarding these complications based on a five-point Likert scale. Part “c” focused on other information related to the surgery, which the researcher assessed with a yes or no response. This included questions about alternative methods, potential risks if the surgery is declined, the consequences and necessary medical interventions post-surgery, providing a phone number or contact method for the surgeon in case of emergencies, mentioning the concept of physician indemnity or waiver of the right to complain against the medical team for potential complications in the IC form, referencing the possibility of obtaining the opinion of the patient or an alternative decision-maker from another physician, and noting whether the patient or alternative decision-maker was given time to think and review the form (a sample of the checklist is included in Appendix 1).

The collected data were analyzed using SPSS v.24 statistical software. Initially, a general description of the dependent variables was performed based on significant independent variables. The frequencies of qualitative variables were reported as ratios (percentages), and quantitative variables were reported as mean ± SD. For data analysis and assessing the relationship between dependent and independent variables, univariate analysis methods such as independent *T*-tests and chi-square tests (depending on the type of variable) or non-parametric equivalents were used. A significance level of less than 0.05 was considered in this study.

To adhere to ethical considerations in this study, after obtaining approval from the ethics committee and receiving the ethics code IR.TUMS.MEDICINE.REC.1399.749, all collected information was kept confidential and analyzed, with results published without mentioning names. It should be noted that the IC waiver was granted by the Research Ethics Committee (REC) and that the data was deidentified and codified for analysis after initial gathering. This study complied with all principles of the Declaration of Helsinki, and a complete explanation of the research objectives and methods was provided in writing and verbally to the officials of the centers and all research units before starting the work, and permission was obtained to use the information from the HIS of the hospital. Additionally, this research did not conflict with the religious, cultural, and social norms of the community.

## Results

**Fig. 1:**
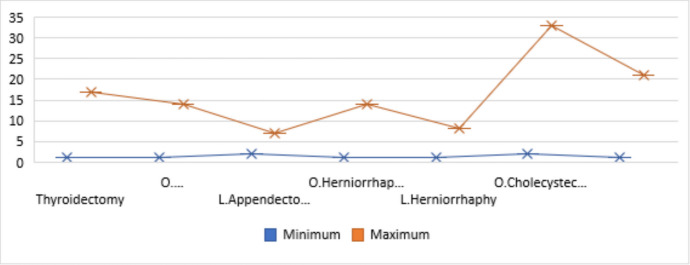
The duration of hospitalization categorized by the type of surgery of the patients studied

In this study, 288 patient records were examined. The mean age of the participants was 47.06 ± 17.73 years (ranging from 15 to 95 years), with 131 individuals (45.5%) identified as female. Regarding marital status, 230 individuals (79.9%) were married. The highest number of patients, totaling 161 individuals (55.9%), was recorded in the city of Tehran (Table 1). The average length of hospital stay was 4.12 ± 4.48 days (ranging from 1 to 33 days), with the longest hospital stay reported for laparoscopic cholecystectomy, averaging 3.67 ± 5.02 days (ranging from 1 to 21 days) (Fig. 1).

**Table 1:** Demographic information of study patients

Demographic information	Number	Percentage (%)
Gender		
Male	157	54.5
Female	131	45.5
Marital status		
Married	230	79.9
Single	58	20.1
Place of residence		
Tehran	161	55.9
Other provincial centers	26	9
County	95	33
Village	4	1.4
Unknown	2	0.7

Two hundred sixty-nine individuals (93.4%) had a consent form recorded in the hospital charts, while 6.4% did not have an informed consent form (ICF) recorded. Among those who had a consent form, 200 individuals (74.3%) were the patients themselves who signed it, and approximately 25% were surrogate decision-makers. The method of confirming the consent provider was accomplished using both signature and fingerprint methods for 221 individuals (82.8%). Additionally, 51.3% of the forms included a date and time. Among the witnesses, 25 individuals (8.6%) were relatives of the patient, while the relationship of the remaining witnesses was not specified. Among the consent providers, general surgery residents represented the largest group, with 265 individuals (98.5%), and confirmation of consent was recorded with a date and time in 61% of cases. A total of 20 forms (6.9%) had pre-operative approval from forensic medicine service, while 8 forms (2.8%) recorded spousal consent. Furthermore, 12 forms (4.5%) contained strike-throughs. None of the forms where the consent provider was someone other than the patient mentioned the reason for not obtaining consent from the patient. There was also no psychiatric consultation to assess the decision-making capacity of the patient in any of the records (Table 2).

**Table 2:** General information of informed consent forms

General information of the consent form	Number	Percentage (%)
Existence of consent form in patient file	269	93.4
Patient’s full name mentioned in the signature	267	92.7
Date and time noted and written by consent provider	138	51.3
Relationship of consent provider to patient		
Self (patient)	200	74.3
Family member	26	9.7
Unknown	43	16
Method of confirming consent provider		
Only signature	11	4.1
Only fingerprint	32	12
Both	221	82.8
Neither	3	1.1
Method of confirming witness		
Only signature	3	1.1
Only fingerprint	10	3.7
Both	43	16
Neither	213	79.2
Title of witness		
Hospital personnel	0	0
Patient’s relatives	25	8.6
Not specified	244	84.72
Name of the individual obtaining consent	256	93.32
Title of individual obtaining consent		
Attending	0	0
Resident	265	92.01
Nurse	0	0
Intern	0	0
Secretary	0	0
Other	0	0
Method of confirming consent provider’s identity		
Signature	4	1.5
Medical stamp	23	8.6
Both	238	88.5
Neither	4	1.5
Others		
Date and time recorded by the consent provider	164	61
Approval of forensic medicine service	20	6.9
Obtaining spousal consent	8	3
Presence of strike-throughs in the informed consent form	12	4.5
Mention of the reason for obtaining consent from others than the patient	0	0
Psychiatric consultation for assessing decision-making capacity	0	0

In Sect. 4 of the checklist, essential information about the nature and method of the surgical procedure was documented in the ICF for only six individuals (2.2%). The comprehensibility of the information provided in the form for an average layperson was rated as completely acceptable for just 12 individuals (4.5%). Notably, the most significant benefit of the surgical procedure was not mentioned in any case, and the completeness of the information regarding the benefits of the surgery received low ratings for all individuals (Table 3).

**Table 3:** Information related to surgical procedures of study patients

Information related to surgical procedures	Number	Percentage (%)
Type of surgery		
Thyroidectomy	50	17.4
Open appendectomy	40	13.9
Laparoscopic appendectomy	8	2.8
Open hernia repair	50	17.4
Laparoscopic hernia repair	40	13.9
Open cholecystectomy	50	17.4
Laparoscopic cholecystectomy	50	17.4
Comprehensibility of information for an average layperson		
Completely acceptable	12	4.5
Acceptable	64	23.8
Intermediate	156	58
Somewhat acceptable	34	12.6
Unacceptable	3	1.1
Mention of the most significant benefit of the surgery	0	0
Completeness of information regarding the benefits of surgery		
Completely comprehensive	0	0
Intermediate	0	0
Comprehensive	0	0
Poor	2	0.7
Completely poor	267	99.3

Among the general complications of surgery, the most frequently mentioned in the consent forms was the risk of wound infection, noted in 241 individuals (89.6%), while the least mentioned was the risk of blood clots in the legs and lungs, noted in 6 individuals (2.2%) (Table 4). In terms of completeness regarding the main general complications, only 2 cases provided fully comprehensive information. Additionally, in the section regarding the completeness of main specific complications, 33 cases provided fully comprehensive information.

**Table 4:** Risk of complications of surgery in study patients

Surgical complications	Number	Percentage (%)
Main general complications of surgery		
Risk of wound infection	241	89.6
Risk of lung infection	47	17.5
Risk of blood clots in legs and lungs	6	2.2
Anesthesia-related respiratory and cardiac complications	160	59.5
Need for ICU admission	69	25.7
Need for blood transfusion	68	25.3
Death during or after surgery due to serious complications	89	33.1
Increased risk of complications in obese and smoking patients	48	17.8
Completeness of information regarding main general complications of surgery		
Completely comprehensive	2	0.7
Comprehensive	91	33.8
Intermediate	24	8.9
Poor	107	39.8
Completely poor	45	16.7
Completeness of information regarding main specific complications of surgery		
Completely comprehensive	33	12.3
Comprehensive	61	22.7
Intermediate	52	19.3
Poor	79	29.4
Completely poor	44	16.4

**Fig. 2:**
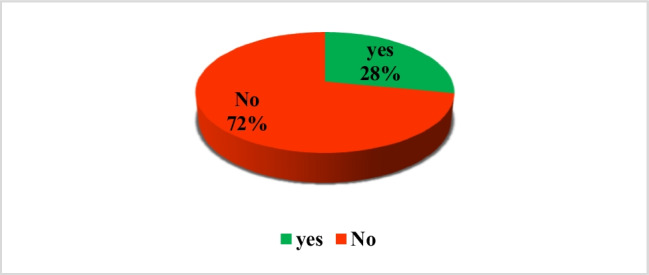
Detailing the risks and necessary medical interventions post-surgery

Other information related to the surgery indicated that in 81 cases (28%), the possible consequences and necessary medical interventions post-surgery were recorded (Fig. 2). None of the forms mentioned the concept of physician indemnity or the waiver of the right to complain against the medical team for potential complications. No patients were provided with a phone number or contact method for the surgeon for emergencies, alternative treatment options, or potential risks if the surgery was declined. Additionally, none of the forms mentioned the possibility of obtaining the opinion of the patient or an alternative decision-maker from another physician, nor did they provide an opportunity for the patient or alternative decision-maker to reflect on and review the content of the form (Table 5).

**Table 5:** Other information regarding the surgery

Other information provided to patients about surgery	Number	Percentage (%)
Alternative methods	0	0
Potential risks if the surgery is declined	0	0
Detailing the consequences and necessary medical interventions post-surgery	81	28
Providing a phone number or contact method for the surgeon in case of emergencies	0	0
Mentioning the concept of physician indemnity or the waiver of the right to complain against the medical team for potential complications in the informed consent form	0	0
Mentioning the possibility of obtaining an opinion from the patient or an alternative decision-maker from another physician	0	0
Mentioning the provision of time for the patient or alternative decision-maker to think about and review the form	0	0
Mention of essential information regarding the nature and method of surgery	6	2.2

The results of this study indicated that, according to statistical analysis using the chi-square test, there was no significant relationship between spousal authorization and gender (*p*-value = 0.089); however, the ratio of spousal authorization in women was nearly three times that of men. Furthermore, the relationship between spousal authorization and the type of surgery was not significant (*p*-value = 0.052), and spousal authorization was recorded only for thyroidectomy, open appendectomy, and laparoscopic cholecystectomy.

In contrast, a significant relationship was found between the ratio of consent providers and the type of surgery (*p*-value = 0.001). This indicates that the type of surgery may influence who provides the informed consent. In this study, the majority of consent providers were the patients themselves. Furthermore, the results of the chi-square test revealed a significant correlation between the existence of a consent form and the type of surgery (*p*-value = 0.018). This suggests that the type of surgery may affect whether a consent form is utilized (Table 6).

**Table 6:** Relationship between provider informed consent and type of surgical procedure

Type of surgery	Patient, *N* (%)	Family, *N* (%)	Unspecified, *N* (%)
Thyroidectomy	38 (76%)	2 (4%)	10 (20%)
Open appendectomy	26 (68.4%)	4 (10.5%)	8 (21.1%)
Laparoscopic appendectomy	7 (87.5%)	0	1 (12.5%)
Open hernia repair	35 (77.8%)	2 (4.4%)	8 (17.8%)
Laparoscopic hernia repair	40 (100%)	0	0
Open cholecystectomy	18 (42.9%)	14 (33.3%)	10 (23.8%)
Laparoscopic cholecystectomy	36 (78.3%)	4 (8.7%)	6 (13%)

**Fig. 3:**
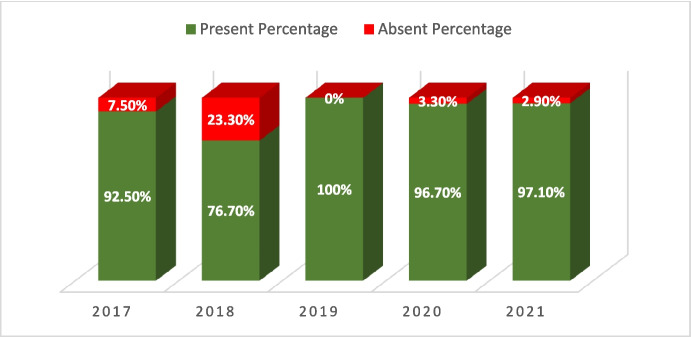
The relationship between the year of hospitalization and the absence of patients’ consent forms

The results also indicated a statistically significant relationship between the year of hospitalization and the absence of a consent form (*p*-value = 0.001). This means that upon reviewing records each year, the number of available consent forms increased; for instance, in 2021, out of 68 cases, 66 patients (97.1%) had a consent form, while 2 patients (2.9%) did not (Fig. 3).

In assessing the completeness of information related to the surgical procedures by type of surgery, it was determined that the highest number of possible complications recorded in IC forms was associated with thyroidectomy, with a total of 178 records of possible complications noted among 50 patients IC forms. This number was higher compared to other types of surgeries. Additionally, the most frequently mentioned complication among the various types of surgeries was the risk of wound infection, with 241 cases reported. Table 7 illustrates the number of possible complications that were recorded in the IC forms for each surgery. The results showed that there was no significant relationship between the type of surgery and the disclosure of general complications (*p*-value > 0.05).

**Table 7:** Surgical complications of study patients by type of surgery

Possible surgical complications	Thyroidectomy	Open appendectomy	Laparoscopic appendectomy	Open herniorrhaphy	Laparoscopic herniorrhaphy	Open cholecystectomy	Laparoscopic cholecystectomy	Total
Risk of wound infection	28	36	8	45	38	40	46	241
Risk of pneumonia	20	2	8	5	0	6	14	55
Risk of blood clots in legs and lungs	2	2	8	2	0	0	0	14
Respiratory, cardiac, and renal anesthesia-related complications	8	22	5	35	24	32	34	160
Need for ICU admission	0	18	3	8	16	12	12	69
Need for blood transfusion	40	4	8	10	6	6	2	76
Death during or after surgery due to serious complications	34	6	1	16	14	10	8	89
Increased risk of complications in obese and smokers	46	0	8	0	2	0	0	56
Total	178	90	49	121	100	106	116	710

## Discussion

This study was conducted to assess the quality of informed consent forms for surgical patients in the General Surgery Department of Shariati Hospital, which is a general metropolitan university hospital in Tehran, Iran. An analysis of patient records from 2017 to 2021 reveals a high adherence rate, with 93.4% of IC forms recorded in patients’ charts before their surgeries. This figure increased to 97.1% by 2021, indicating a positive trend. These results are consistent with earlier studies that show an upward trajectory in obtaining IC, reflecting the broader changes in Iran’s healthcare system as it increasingly emphasizes the importance of this practice (Joolaee et al. 2017; Sheikhtaheri and Farzandipour 2010). Various factors at different levels may have contributed to this change, including significant social and cultural transformations in Iranian society over recent decades. These shifts encompass a trend toward secularization, evolving patterns of religiosity among the population, and a gradual move toward a less conservative society (Godazgar and Mirzaei 2025). Among others, one influential factor contributing to this increase could be the fact that the availability and presence of informed consent forms in patients’ charts is an important criterion for hospital accreditation, commonly assessed by evaluators of the national hospital accreditation system. These evaluations are crucial for hospitals’ reputations and economic status, as higher-ranked hospitals are eligible to receive higher tariffs (Moosavi et al. 2022).

A particularly noteworthy finding is the shift toward a more individualistic approach, with 74.3% of consents being provided directly by patients, a significant increase from 55% reported in a 2010 study (Sheikhtaheri and Farzandipour 2010). This change marks a shift away from traditional norms in many Low-and-Middle-Income Countries (LMICs) and Eastern cultures, where family members often play a dominant role in decision-making, suggesting a cultural transition toward recognizing and empowering patient autonomy. In comparison, studies in Turkey and Pakistan show similar collectivist backgrounds, though their paths differ. In Turkey, family involvement in decision-making remains strong, often prioritizing relatives’opinions over those of the patients (Kara 2007). Meanwhile, in Pakistan, decisions regarding consent are largely driven by family, indicating a slower movement toward individualism (Jafarey and Farooqui 2005). Iran’s healthcare system is undergoing a meaningful ethical shift, gradually moving away from family-centered decision-making traditions to prioritize patients’ own voices in medical consent. This evolution reframes care by centering personal choice over inherited norms, empowering individuals to shape their healthcare journeys. Despite the role of patients and their families in such a shift, the role and attitude of healthcare professionals are also important. They can emphasize the importance of involving patients themselves in the informed consent (IC) process. This study revealed that almost all ICs were obtained by general surgery residents who are mostly young physicians in their third or early fourth decades of life and belong to the younger, modernized generation of Iranians. Additionally, the study includes patients who underwent elective surgeries. However, the practices and results might differ for more sensitive health conditions such as end-of-life decision-making, cancer treatments, and similar situations.

In this study, the requirement for spousal consent in only 2.8% of cases further underscores this shift in practice. In a few available studies in Iran, spousal authorization and approval were sought in 69% of cases involving women, even though ethical guidelines affirmed their autonomy. In those situations, consent was only obtained from the woman in 19.7% of cases (Jarayedi and Asghari 2018). The 2018 Code of Ethics issued by Iran’s Medical Council reinforces this change by stating that third-party consent is unnecessary for competent women. This landmark document explicitly declares that spousal consent is not required for medical procedures involving women, including elective surgeries like cosmetic enhancements. Article 73 of the Code specifically addresses third-party authorization for IC, emphasizing that requiring permission from husbands or fathers for any medical intervention is not ethically justified when a woman is competent to make her own decisions (Shamsi-Gooshki et al. 2020). The diminished reliance on spousal authorization and consent reflects evolving sociocultural dynamics in Iran, such as increased educational attainment and economic autonomy among women—factors that have bolstered their agency in advocating for rights. This shift further resonates with recent strides in women’s rights movements across the country (Tohidi 2016). In contrast, studies in Pakistan indicate that physicians still prioritize spousal consent for married women (Jafarey and Farooqui 2005), and in Nigeria, nearly half of women reported needing their husband’s approval to participate in research (Osamor and Kass 2012).

A distinctive element of the IC process in this study is the widespread use of witnesses, with 93.4% of forms bearing witness confirmation, of which 8.6% were explicitly identified as patient relatives and none were hospital staff. Witness confirmation has reached an impressive 93.4%, marking a considerable increase from 77.6% in emergency cases and 59.9% in non-emergency cases back in 2008, demonstrating a growing reliance on external validation. Involving witnesses in the IC process, which typically verifies the consent provided by the patient or surrogate, on the one hand, could enhance procedural transparency and probably reflects a culturally embedded practice by including other influential members of the family, which was seen as a prevalent practice in this study’s healthcare settings. This stands in sharp contrast to Western IC protocols, where direct patient-physician agreement predominates, and third-party witnesses are seldom required unless the patient lacks capacity from a bioethical perspective. While someone may argue that this practice could strengthen non-maleficence by documenting the process and mitigating coercion risks, it creates other critical ethical challenges. The result of this study shows that this is a standard practice and possibly a hospital internal policy, at least in this specific university hospital, despite the fact that none of the laws or national regulations require healthcare providers to obtain witness signatures for IC. This routine practice could be a risk to confidentiality. The involvement of relatives as witnesses risks exposing sensitive medical information to family members, potentially violating patients’ rights to privacy—a direct contradiction of the ethical duty to protect personal health data. Although, as mentioned before, in this study, spousal authorization of informed consent was not a dominant pattern, with less than 3% of informed consent forms having explicit authorization from spouses, the prevalent involvement of witnesses other than hospital staff could be another way of giving a role to individuals other than patients. This practice could exacerbate the already existing power dynamics within families. Furthermore, reliance on unspecified witnesses (rather than hospital staff) complicates oversight, balancing transparency with risks to discretion. Literature suggests that impartial third-party witnesses could mitigate potential bias, a view reinforced by a 2023 study advocating for neutrality in witnessing mechanisms to uphold ethical integrity (Pai et al. 2023). The role of other influential actors in involving family members in the informed consent process, even in the absence of a legal or regulatory requirement, should not be overlooked. Defensive motivations could be another reason for including family members as witnesses in the informed consent process to avoid or reduce the risk of future lawsuits. Recent studies show a high prevalence of defensive medicine in Iran (Eftekhari et al. 2023), and qualitative evidence from Iranian contexts indicates that over-documentation could be a form of defensive medicine (Shamsi Gooshki 2024).

A longitudinal comparison with a 2008 study by Hajavi et al. sheds light on notable shifts in the IC process over the past 15 years, reflecting an evolving ethical and legal landscape in Iranian healthcare (Hajavi et al. 2008). In the current study, 4% of consent forms showed strike-throughs, a slight decrease from 5.4% in 2008. Additionally, only 0.7% of forms omitted patients’ full names, which is a significant improvement from 7.2% previously, indicating a rise in administrative accuracy. However, 16% of cases still left the consent provider’s relationship unspecified, highlighting a gap that continues to hinder transparency.

Another significant shift is around the issue of “waiving the patient’s right to legal complaints and filing lawsuits.” Despite the fact that even signing such a waiver form by patients cannot eliminate their rights to legal complaints, this practice was prevalent and a part of the IC form for decades in Iran. It probably worked for individuals who were not familiar with their fundamental right to file complaints, which could not be waived by signing such a document. However, healthcare providers and institutions might have thought that this approach could reduce the possibility of lawsuits, particularly from patients with lower levels of legal literacy. This could explain the high prevalence of using such waivers, which were present in 96.2% of the records from 2008. Interestingly, this study shows that the use of such content in the hospital’s IC forms has completely disappeared (0%) during the study period. This positive shift away from defensive legal practices toward a more patient-centered approach to consent enhances patient autonomy by freeing them from preemptive liability waivers and positioning them as active decision-makers rather than passive subjects of institutional protection. However, it is not possible to generalize this finding to other health institutions and facilities in Iran. Shariati Hospital is one of the largest university hospitals with a well-functioning hospital ethics committee during the years of this study, and it also established one of the first clinical ethics consultation services during the same period.

The quality of information shared with patients in this study reveals significant deficiencies that undermine the ethical foundation of IC, specifically regarding the disclosure and understanding elements. Only 2.2% of patients received essential details about the nature and process of surgical procedures. For 74% of those who signed consent forms, critical elements—such as prognosis, alternative treatments, and risks of refusal—were left entirely unaddressed. Most alarmingly, no forms communicated the primary benefits of surgery, with 99.3% rated completely poor for completeness of benefit-related information. Furthermore, only 0.7% and 12.3% achieved fully comprehensive disclosure of general and specific complications, respectively. Additionally, just 28% of patients received adequate post-surgical care information, and none were provided with emergency contact details, alternative options, or insights into physician indemnity. These critical omissions directly violate autonomy by denying patients the essential information required to guide their own healthcare decisions (Beauchamp 2011). When consent forms exclude treatment benefits, alternatives, and risks, patients are left without the tools to exercise meaningful choice, effectively trapped in a one-sided narrative. The absence of information about potential benefits in the content analysis of the IC forms could also be explained by a defensive attitude toward the informed consent process by healthcare providers. Since courts generally place more emphasis on the lack of information about risks rather than benefits in potential lawsuits, healthcare providers may have prioritized documenting risks over benefits to protect themselves legally. Compounding this issue and the lack of dedicated time for reflection and questioning denies patients the opportunity to deliberate, reducing consent to a rushed formality rather than an informed, voluntary act. Despite this, one might argue that most of the communication during the informed consent process in Iranian clinical culture remains verbal. Even if IC forms lack sufficient information for a valid consent process, much of the required information could be delivered verbally, outside the documentation in the IC forms. Another important observation is the absence of information sheets, which could serve as a useful complementary tool to the IC process. These sheets are standardized bulletins or brochures that provide a wide range of information about typical medical procedures routinely performed in a healthcare facility, such as the surgeries targeted in this study. These template information sheets could be provided to patients, even at the time of admission for elective surgeries. This approach would allow patients enough time and opportunity to read, understand, and seek advice regarding the procedures.

These findings suggest that legal concerns and defensive motivations outweigh ethical and moral considerations, such as respecting patients’ autonomous choices and providing them with the opportunity to exercise their moral agency. This tension between legal compliance and ethical care in informed consent becomes glaringly clear in practices like fingerprinting—used in 82.8% of cases—and some cases of seeking advice from forensic medicine services (6.9%). According to a general assumption, fingerprints provide a more reliable legal guarantee than signatures because people may later deny their signature in the case of a lawsuit or use a fake signature. However, fingerprints cannot be easily denied. This specific practice of requiring fingerprints could be seen as another indication that the informed consent process is being used more as a legal protective tool and defensive measure, rather than as an ethical construct. While healthcare providers may think that fingerprints, typically added alongside signatures, create a paper trail to shield doctors from lawsuits, they are not related in any way to the ethical underpinnings of IC and do nothing to ensure patients actually understand or voluntarily agree to treatment. These findings mirror a troubling pattern in Iran’s healthcare system, where fear of legal consequences pushes clinicians toward excessive paperwork—like mandatory fingerprints—as a defensive medicine (Eftekhari et al. 2023).

Similarly, requesting forensic medicine opinions is another instrument to protect hospitals and healthcare providers more than patients. In the absence of efficient legal departments in hospitals, forensic medicine specialists are usually consulted, particularly in metropolitan university hospitals where forensic medicine services are available, especially in those with residency programs for forensic medicine as well as big private hospitals. As part of the judiciary system in the country, the Legal Medicine Organization of Iran and its expert committees provide opinions to the courts in cases of medical complaints. Healthcare providers may perceive these expert opinions as a protective shield, either due to their knowledge of the law (despite the fact that legal training is only minimal in residency curricula) or because they believe that having such consultations available will influence the expert committees and, consequently, court decisions in their favor. However, we view this practice as a potential risk to the clinical environment, shifting the patient-physician relationship from one based on mutual trust and the virtue of care to one dominated by legal concerns. The involvement of forensic medicine services in the informed consent process could be one of the sources of the highly prevalent defensive practices in Iran’s healthcare system. The potential conflict of interest for forensic medicine specialists, who may benefit from a defensive environment due to the fee-for-service reimbursement scheme in Iranian hospitals that increases their income when the number of such consultations increases, furthers these concerns. In essence, by requesting such expert opinions, healthcare professionals and health institutions prioritize institutional safety nets over meaningful patient-provider communication, reducing consent to a bureaucratic checkbox rather than a tool for empowerment. This issue is even more concerning because, in most cases, the financial burden of seeking such consultations falls on the patients themselves in private hospitals or is shared between public insurance and the patients in public hospitals.

Despite the fact that the COVID-19 pandemic offers a critical lens to assess its influence on IC processes, data stratification reveals a notable increase in consent form availability from 93.4% overall to 97.1% in 2021, which could be attributed to heightened administrative scrutiny during the pandemic bolstered procedural compliance, possibly as a safeguard because of the increased risk of being infected by the SARS-CoV-2 virus as a result of hospitalization for elective surgeries during the pandemic. However, the impact of the pandemic on the practice in IC requires further research.

Understanding the provided information by patients is another crucial component of a valid and ethically acceptable IC. The comprehensibility and understandability of consent forms in this study present a significant barrier, with only 4.5% rated fully understandable by laypersons, revealing a substantial ethical shortcoming in facilitating informed decision-making. In comparison, studies in other contexts have shown higher levels of comprehension; for example, 94.1% of patients understood the information provided in a similar study in Navi Mumbai (Patil et al. 2023), and in Nepal (Basukala et al. 2023), 95.4% found it understandable, although gaps persisted in specific details, such as benefits or alternatives. Earlier studies within Iran also depict a troubling picture: a 2012 investigation revealed poor patient knowledge despite a consent rate of 44.9% (Tahereh Khazaei et al. 2012), while a 2014 study reported that only 19.5% found forms comprehensible, with scant details on diagnosis, treatment options, or risks (Nafiseh Zafarghandi and Fatemeh Sarv 2014). This enduring lack of clarity directly violates autonomy by preventing patients from making informed choices, a core requirement of ethical IC. It also undermines beneficence, as patients cannot assess the value of procedures for their well-being without understandable information (Faden and Beauchamp 1986; Beauchamp 2011). Again, this systemic failure may reflect a process still focused on legal compliance over genuine engagement, necessitating comprehensive reform to align IC with ethical standards.

A striking evolution in this study lies in who obtain IC, with 98.5% of forms now obtained by residents—a significant change from findings of another study 15 years ago, where ward secretaries, non-clinical staff, handled all (Taghaddosinejad et al. 2008). Residents’ direct involvement in care fosters richer dialogue, enhancing patient autonomy as they engage with a knowledgeable intermediary rather than an administrative proxy—an approach that patients are more likely to trust for addressing concerns and clarifying implications (Shah et al. 2024). A 2023 scoping review supports this trend, noting that surgical assistants are increasingly taking on this role across various contexts, reflecting a global move toward professionalizing IC (Gardiner et al. 2023). This shift marks an ethical pivot, elevating IC from bureaucratic formality to a practice rooted in informed trust. Replacing clerical staff with residents’ honors autonomy by ensuring explanations come from those equipped to link clinical knowledge to individual needs, ultimately prioritizing meaningful understanding over procedural compliance. Although the involvement of residents in the informed consent process can be seen as a step forward, the absence of attending physicians in this process is a critical shortcoming. As mentioned earlier, patients expect their surgeon to discuss the information with them. Another concern is the general public assumption that in university and teaching hospitals, residents and learners do everything, which may exacerbate concerns regarding the active participation of attending surgeons in the surgery itself. This assumption is prevalent among Iranian patients, who often believe that this is the case in university hospitals. Another concern about the high level of involvement of residents is the growing worry regarding their heavy workload, burnout, and the possibility of exploitative behaviors toward them. These issues have contributed to several recent cases of suicide among medical residents in Iran in the past few years.

The results of this study revealed no signs of coercion. While the voluntariness of consent could not be directly evaluated, involuntary practices are unlikely due to the elective nature of the surgical interventions studied. Similarly, there were no signs of active evaluation of decision-making capacity, as no psychiatric consultations were requested for patients. Considering the age of some patients, which was relatively high, it is unlikely that none of them had issues with their capacity and competency for decision-making. The lack of any indication of such an evaluation, however, is an alarming concern in this regard (Table 8).

**Table 8:** Summary of key discussion points

Point	Summary
High adherence to obtaining IC and availability of forms in patients records	In 93.4% of patients/records, IC forms were available, up to 97.1% in 2021, showing procedural improvement
Significant gap regarding the crucial elements of valid IC	IC forms suffer from a significant deficit in disclosure and understanding elements of IC. Only 2.2% of patients received essential details about the nature and process of surgical procedures and only 4.5% of IC forms rated fully understandable
Precedence of legal concerns that outweigh ethical and moral considerations	Presence of defensive elements such as 93.4% witnessed, near 83% recorded fingerprints alongside with signatures, and approximately 7% requested forensic medicine advice
Improvement regarding requiring third-party authorization	Spousal authorization of patients’ consent was only seen in 2.8% of records
Professional involvement in IC process	Residents obtained 98.5% of consents

## Limitations

There are several limitations that should be considered when interpreting and generalizing the results of this study. This study was conducted in a metropolitan general university hospital in Tehran, the capital city of Iran, which is publicly funded, relies heavily on residents as a major part of its workforce, and has an active hospital ethics committee. These factors limit the ability to generalize the findings to other settings, such as other cities and small towns, regions of the country with specific ethnic or cultural contexts, non-teaching hospitals, private hospitals, or those with little to no clinical ethics support services. Another important limitation is that the data pertains to specific elective surgical procedures, and caution should be exercised when generalizing these findings to other medical interventions.

## Conclusion

This study reveals significant gaps in the practice of informed consent at this specific hospital, which, in some respects, may be more or less generalizable to the broader situation in the country, given the study’s limitations. The results highlight gaps in various elements necessary for a valid informed consent process, particularly in disclosure and understanding. However, we cannot draw conclusions about voluntariness and the assessment of decision-making capacity as other key elements of informed consent. In this study, we concluded that IC forms prior to surgical procedures do not fully and accurately address all required aspects, which are essential for a valid ICF, including those related to the surgery and post-operative care to patients, such as risks, benefits, possible complications, and alternative treatment. Despite these findings that undermine patients’ autonomous decision-making, this study provides positive evidence, such as increased involvement of healthcare team members in the informed consent process and a significant decrease in the explicit requirement for third-party authorizations, particularly for female patients.

A major part of the findings provides insights into the precedence of legal concerns that outweigh ethical and moral considerations in the informed consent process, using it more as a tool for self-defense rather than respecting patients’ rights to autonomous decision-making. However, even such defensive approaches have shifted from the explicit requirement for patients to sign documents that indemnify physicians or waive their right to complain against the medical team in case of potential complications, toward other, more soft but implicit forms, such as recording fingerprints and requiring witnesses for almost all informed consent forms.

## Recommendations

Based on the findings of this research, we can recommend the following:

- Expanding conducting further research on IC practices in various healthcare settings, including non-teaching hospitals, private facilities, and regions with specific cultural or ethnic contexts, to gain a comprehensive understanding of its application across diverse environments.
- Implementing standardized information sheets or brochures that explain common medical procedures. These should be available to patients, especially at the time of admission for elective surgeries, giving them ample time to understand and make informed decisions.
- Investigating and addressing the defensive motivations behind IC practices that may undermine the ethical essence of the process. It is crucial for authorities to identify the causes of these defensive behaviors and implement strategies to reduce them.
- Providing comprehensive training for healthcare professionals and health facilities administrators on valid IC consent processes.

## Supplementary Information

Below is the link to the electronic supplementary material.

Supplementary file1 (DOCX 18 KB)

## Data Availability

The data are available from the corresponding author on reasonable request.
