# Supplementary material for: Gaps and Gains in Informed Consent for Surgery in a Non-Western Context: Beyond the Signatures of Iranian Patients
Source: Asian Bioeth Rev. 2025 Nov 17;18(2):239–63. doi: 10.1007/s41649-025-00372-2 (PMC13076807; doi:10.1007/s41649-025-00372-2)
Supplement: Supplementary file 1 — Supplementary file1 (DOCX 18 KB) [file 41649_2025_372_MOESM1_ESM.docx]

**Checklist for Thyroidectomy**

**In the Name of God**
**School of Medicine, Tehran University of Medical Sciences**

Information Collection Form for the Thesis Titled:
“Evaluating the Quality of Informed Consent Forms for Surgical Patients in the General Surgery Department of Shariati Hospital from 2009 to 2019”
Ethics Code: IR.TUMS.MEDICINE.REC.1399.749

**Section 1: Demographic Information of the Patient**

1-1) Patient Number: __________________
1-2) Age: __________________
1-3) Gender:
     Female ○
     Male ○
1-4) Address of Residence:
     Tehran ○
     Other Provincial Centers ○
     County ○
     Village ○
     Unknown ○
1-5) Insurance Status:
     Unknown ○
     None ○
     Armed Forces ○
     Social Security ○
     Medical Services ○
     Rural ○
     Health ○
     Other Insurances ○
1-6) Marital Status:
     Married ○
     Single ○

**Section 2: Information Related to the Disease**

2-1) Type of Disease: __________________
2-2) Length of Hospital Stay: __________ days

**Section 3: General Information on the Informed Consent Form**

3-1) Is the informed consent form present in the patient’s file?
     Yes ○
     No ○
3-2) Is the patient’s full name mentioned?
     Yes ○
     No ○
3-3) Relationship of the consent provider to the patient:
     The patient themselves ○
     Family member ○
     Unknown individual ○
3-4) Is the date and time recorded by the consent provider?
     Yes ○
     No ○
3-5) Method of confirming the consent provider:
     None ○
     Signature ○
     Fingerprint ○
     Both ○
3-6) Witness Confirmation:
     None ○
     Signature ○
     Fingerprint ○
  &nbsp
     Not specified ○
     Patient’s relative ○
     Hospital personnel ○
     Other ○
3-8) Is the name of the individual obtaining consent mentioned?
     Yes ○
     No ○
3-9) Title of the individual obtaining consent:
     Attending ○
     Resident ○
     Nurse ○
     Intern ○
     Secretary ○
     Other ○
3-10) Method of confirming the consent receiver:
     None ○
     Signature ○
     Stamp ○
     Both ○
3-11) Is the date and time recorded by the consent receiver?
     Yes ○
     No ○
3-12) Forensic Confirmation:
     Yes ○
     No ○
3-13) Was spousal consent obtained?
     Yes ○
     No ○
3-14) Is there any strike-through in the informed consent form?
     Yes ○
     No ○
3-15) In cases where the consent provider is someone other than the patient, is there a mention of the reason for not obtaining consent from the patient?
     Yes ○
     No ○
3-16) Was a psychiatric consultation or similar conducted to assess the decision-making capacity of the patient?
     Yes ○
     No ○

**Section 4: Information Related to the Surgical Procedure**

4-1) Type of Surgery:
     Thyroidectomy
4-2) Is there mention of essential information regarding the nature and method of the surgical procedure?
     Yes ○
     No ○
     *Complete removal of the thyroid gland or part of it through an incision along the neck.*
4-3) Comprehensibility of the information in the form for an average layperson: (1 to 5)
     5 - Completely Acceptable
     4 - Acceptable
     3 - Intermediate
     2 - Somewhat Acceptable
     1 - Unacceptable
4-4) Is the most significant benefit of undergoing the surgery mentioned?
     Yes ○
     No ○
     - Removal of cancerous gland to prevent disease progression
     - Removal of glands causing hyperthyroidism due to their secretions
     - Reduction of disease symptoms
     - Removal of an enlarged thyroid that exerts pressure on the airway
*Completeness of information regarding the benefits of the surgery: (1 to 5)*
     5 - Completely Comprehensive
     4 - Comprehensive
     3 - Intermediate
     2 - Poor
     1 - Completely Poor
4-5) Is there mention of the main general complications of the surgery?
     Yes ○
     No ○

1. Risk of wound infection
        Yes ○
        No ○
2. Risk of lung infection
        Yes ○
        No ○
3. Risk of blood clots in legs and lungs
        Yes ○
        No ○
4. Respiratory and cardiac anesthesia complications
        Yes ○
        No ○
5. Possibility of needing ICU admission
        Yes ○
        No ○
6. Need for blood transfusion
        Yes ○
        No ○
7. Death during or after surgery due to serious complications
        Yes ○
        No ○
8. Increased risk of complications in obese and smoking patients
        Yes ○
        No ○
   *Completeness of information regarding the main general complications of the surgery: (1 to 5)*
        5 - Completely Comprehensive
        4 - Comprehensive
        3 - Intermediate
        2 - Poor
        1 - Completely Poor
   4-6) Is there mention of the main specific complications of the surgery?
        Yes ○
        No ○
9. Injury to large neck vessels and nerves in the neck area
        Yes ○
        No ○
10. Injury to the parathyroid glands (finger tingling and spasms in hands and feet) and long-term need for calcium
         Yes ○
         No ○
11. Bleeding in neck tissues and pressure on the respiratory tract
         Yes ○
         No ○
12. Injury to vocal cords (hoarseness and reduced pitch)
         Yes ○
         No ○
13. Injury to the trachea and esophagus
         Yes ○
         No ○
14. Cardiac and pulmonary complications
         Yes ○
         No ○
15. Need for reoperation after pathology results are ready
         Yes ○
         No ○
16. Non-healing of the wound and formation of painful scars
         Yes ○
         No ○
    *Completeness of information regarding the main specific complications of the surgery: (1 to 5)*
         5 - Completely Comprehensive
         4 - Comprehensive
         3 - Intermediate
         2 - Poor
         1 - Completely Poor
    4-7) Are alternative methods mentioned?
         Yes ○
         No ○
         *Radioactive iodine therapy in specific cases*
    4-8) Are potential risks mentioned in case of refusing the surgery?
         Yes ○
         No ○

- Increased disease symptoms
- Increased size of the thyroid gland
- Increased cancerous mass
- Enlargement of neck tissues
- Pressure on the airway and breathing difficulties
- Metastasis of cancer cells to lymph nodes and other organs
- Mortality
  4-9) Are the consequences and necessary medical interventions post-surgery described?
       Yes ○
       No ○
  4-10) Is there a phone number or contact method for the surgeon in case of emergencies?
       Yes ○
       No ○
  4-11) Is there mention of the physician’s indemnity or waiver of the right to complain against the medical team for potential complications in the informed consent form?
       Yes ○
       No ○
  4-12) Is there mention of the possibility of obtaining the opinion of the patient or an alternative decision-maker from another physician?
       Yes ○
       No ○
  4-13) Is there mention of giving the patient or alternative decision-maker an opportunity to think and review the form?
       Yes ○
       No ○
